# Case of Fracture of Both Clavicles

**Published:** 1850-01

**Authors:** Wm. Quail

**Affiliations:** Pittsburg


*Case of Fracture of both Clavicles.* By Wm. Quail, M. D., of Pittsburg. (Communicated by Prof. Mütter.)

On the 13th of last month I was called to treat a case of fracture of both clavicles. The man, a stout, healthy Irishman, æt. 54, in attempting to control a cart horse, was crushed between one of the wheels and a bank, and the consequence was, fracture of both bones at the scapular ends.

Not having previously seen or read of such an accident, my embarrassment, as you can readily imagine, was great. All the different methods suggested by surgeons for fractured clavicle, I thought of at once. My embarrassment arose from the fact of not having a sound shoulder to operate upon.

I finally concluded to try the simple and efficient apparatus of Fox. I had two pads, and two elbow pieces prepared. I made the two collars in the form of half rings, attaching each end to the pad, thus serving the purpose of fixing the pads in position, and as collars to which might be fastened the tapes of each elbow piece. I applied the apparatus as thus modified, using the ordinary sling to support the hands, and it has answered admirably; indeed, I cannot imagine how else it could be managed.

The patient walked about the house during the whole time, and suffered comparatively little or no pain. To-day I removed the apparatus. There is no deformity which time will not cure.

The case was seen the next day after reduction, by my friend, Dr. Black, and by Mr. McDonald, one of my fellow students of Jefferson Medical College.

What would I have done with Dessault's three single-headed rollers, eight yards long, &c., and doubled, too, in the hot months of July and August ?

